# Liver Ischemia and Reperfusion Induce Periportal Expression of Necroptosis Executor pMLKL Which Is Associated With Early Allograft Dysfunction After Transplantation

**DOI:** 10.3389/fimmu.2022.890353

**Published:** 2022-05-17

**Authors:** Shaojun Shi, Eliano Bonaccorsi-Riani, Ivo Schurink, Thierry van den Bosch, Michael Doukas, Karishma A. Lila, Henk P. Roest, Daela Xhema, Pierre Gianello, Jeroen de Jonge, Monique M. A. Verstegen, Luc J. W. van der Laan

**Affiliations:** ^1^ Department of Surgery, Erasmus MC Transplant Institute, University Medical Center, Rotterdam, Netherlands; ^2^ Abdominal Transplant Unit, Cliniques Universitaires Saint-Luc, Université Catholique de Louvain, Brussels, Belgium; ^3^ Pôle de Chirurgie Expérimentale et Transplantation Institute de Recherche Expérimentale et Clinique, Université Catholique de Louvain, Brussels, Belgium; ^4^ Department of Pathology, Erasmus MC-University Medical Center, Rotterdam, Netherlands

**Keywords:** ischemia-reperfusion injury, programmed cell death, non-parenchymal cell, myofibroblast, liver transplantation

## Abstract

**Background:**

Early allograft dysfunction (EAD) following liver transplantation (LT) remains a major threat to the survival of liver grafts and recipients. In animal models, it is shown that hepatic ischemia-reperfusion injury (IRI) triggers phosphorylation of Mixed Lineage Kinase domain-like protein (pMLKL) inducing necroptotic cell death. However, the clinical implication of pMLKL-mediated cell death in human hepatic IRI remains largely unexplored. In this study, we aimed to investigate the expression of pMLKL in human liver grafts and its association with EAD after LT.

**Methods:**

The expression of pMLKL was determined by immunohistochemistry in liver biopsies obtained from both human and rat LT. Human liver biopsies were obtained at the end of preservation (T0) and ~1 hour after reperfusion (T1). The positivity of pMLKL was quantified electronically and compared in rat and human livers and post-LT outcomes. Multiplex immunofluorescence staining was performed to characterize the pMLKL-expressing cells.

**Results:**

In the rat LT model, significant pMLKL expression was observed in livers after IRI as compared to livers of sham-operation animals. Similarly, the pMLKL score was highest after IRI in human liver grafts (in T1 biopsies). Both in rats and humans, the pMLKL expression is mostly observed in the portal triads. In grafts who developed EAD after LT (n=24), the pMLKL score at T1 was significantly higher as compared to non-EAD grafts (n=40). ROC curve revealed a high predictive value of pMLKL score at T1 (AUC 0.70) and the ratio of pMLKL score at T1 and T0 (pMLKL-index, AUC 0.82) for EAD. Liver grafts with a high pMLKL index (>1.64) had significantly higher levels of serum ALT, AST, and LDH 24 hours after LT compared to grafts with a low pMLKL index. Multivariate logistical regression analysis identified the pMLKL-index (Odds ratio=1.3, 95% CI 1.1-1.7) as a predictor of EAD development. Immunohistochemistry on serial sections and multiplex staining identified the periportal pMLKL-positive cells as portal fibroblasts, fibrocytes, and a minority of cholangiocytes.

**Conclusion:**

Periportal pMLKL expression increased significantly after IRI in both rat and human LT. The histological score of pMLKL is predictive of post-transplant EAD and is associated with early liver injury after LT. Periportal non-parenchymal cells (i.e. fibroblasts) appear most susceptible to pMLKL-mediated cell death during hepatic IRI.

## Introduction

Liver transplantation (LT) is the only curative intervention for patients with end-stage liver diseases or hepatic malignancies (1). Procurement of the liver graft is associated with hepatic ischemia-reperfusion injury (IRI) in the donor, during storage and transportation, and after transplantation in the recipient. Though the extent of IRI varies per graft, overall, it has a negative impact on LT outcome (2). Early allograft dysfunction (EAD), defining the initial inferior function of the implanted liver is a critical determinant of graft survival and recipient outcome following both cadaveric and living donor LT (3–5). Risk factors associated with EAD development after LT include donor and recipient characteristics (age, BMI, and lifestyle), and intraoperative events such as prolonged cold or warm ischemia time, macrovesicular steatosis, and intra-operative transfusion requirements, and prolonged operation time (6–8). Secondary to IRI, these combined factors contribute to EAD by inducing hepatocellular damage, oxidative stress, accidental or programmed cell death, and severe inflammatory responses, which were clinically observed and confirmed in histological studies (7, 9–12).

The two major types of cell death caused by IRI are apoptosis and necrosis. This has been extensively studied in both animal models and human tissue biopsies (13). Gujral et al. (14) demonstrated that hepatocyte death in IRI was mainly caused by necrosis, especially during the reperfusion stage. Only a small population of sinusoidal endothelial cells and hepatocytes underwent apoptosis. Another study described that apoptosis in hepatocytes found in donor liver biopsies collected at the end of organ procurement predicted EAD after LT (10). However, studies unraveling the role of the particular types of cell death in the development of EAD are scarce.

Recent studies have shown that liver cells affected by IRI, including parenchymal and non-parenchymal cells, could die not only as a result of apoptosis or necrosis but also by several non-apoptotic forms of programmed cell death, also known as “regulated necrosis” (15). Of those, necroptosis is one of the most studied and incorporates the characteristics of both apoptosis and necrosis (16). Necroptosis and apoptosis share the same upstream mechanism which is induced by cell death receptors. Upon the inhibition of caspase 8 or Fas-associated *via* death domain, the complex of receptor-interacting protein kinase 1 (RIPK1) and RIPK3 is formed and switches the apoptosis machinery into necroptosis. The mixed lineage kinase domain-like protein (MLKL) is phosphorylated and oligomerized subsequently, translocating to the cell membrane and mediating the cell rupture to execute necroptosis (17). Although pMLKL has been widely regarded as the hallmark of necroptosis, the activation of pMLKL has been observed in endoplasmic reticulum stress-related apoptosis (18), hinting that pMLKL-mediated cells death might not be not exclusively necroptosis. In the case of pMLKL-mediated necroptosis, the leakage of the damage-associated molecular patterns from ruptured cells further contributes to inflammatory response, also known as sterile inflammation or necroinflammation, which is a critical pathological process during hepatic IRI.

The emerging role of necroptosis in hepatic IRI has been reported in a few experimental studies. Based on murine IRI models, necroptosis has been found to not only result in hepatic damage during IRI in healthy livers (19, 20) but also aggravate IRI in both aging (21) and steatotic (22, 23) livers. On the contrary, there are also studies demonstrating that necroptosis does not play a critical role in murine hepatic IRI (24, 25). This contradiction may arise from the difference in the animal model used. Of note, the necroptosis machinery varies between species and can therefore lead to a potential discrepancy in experimental and clinical studies (15, 26). However, clinical evidence of the involvement of necroptosis mediators, such as pMLKL, in human liver IRI is lacking.

We have previously shown that necroptosis is involved in various human liver diseases in an etiology-dependent manner (27). Interestingly, although based on only a few cases, we found extensive expression of pMLKL in human liver biopsies during LT, implying the potential existence of pMLKL-mediated cell death in human liver IRI. The pMLKL expression as previously published was mostly found in the portal triad area, which was different from that in other biopsies obtained from patients with chronic liver diseases. The portal triad consists of the bile duct, hepatic artery, and portal vein, supported by numerous non-parenchymal cells with distinct molecular features (28). Myofibroblasts represent one of the major stromal and extracellular matrix (ECM) producing cells in the portal triads, and originate from hepatic stellate cells (HSCs), portal fibroblasts (PFs), and fibrocytes, which serve a variety of functions in the response to acute and chronic insults (29–31). We hypothesized that the non-parenchymal cells, possibly the myofibroblasts, present in the portal triads might be the major cell population co-expressing pMLKL during liver transplantation. In the present study, we aim to investigate the pMLKL expression in both rat and human liver biopsies and whether the histological score of pMLKL is associated with the development of EAD after transplantation. Furthermore, we characterized the molecular phenotype of the liver cells expressed pMLKL.

## Patients and Methods

### Patient Selection and Data Collection

We performed a retrospective study using liver biopsies from patients undergoing orthotopic liver transplantation (OLT) between April 2008 and March 2012 at the Erasmus University Medical Center, Rotterdam, the Netherlands. Of 83 cases enrolled in our previous prospective study (32), 64 patients were included in this study. Due to incomplete biopsy retrieval from the pathology biobank, 19 patients were excluded. The main demographic and clinical characteristics of included recipients and donors were listed in **Table S1**. Postoperative laboratory data were collected for the first 7 days after OLT. This study was approved by the Erasmus MC medical ethics council (MEC-2014-060). All patients gave informed consent for the use of material for research purposes.

### Definitions of Post-Transplant Complications

Early allograft dysfunction (EAD) was defined according to the criteria of Olthoff et al. (33) by the presence of one or more of the following: (i) bilirubin ≥ 10 mg/dL on a postoperative day (POD) 7; (ii) INR ≥ 1.6 on POD7; (iii) alanine aminotransferase (ALT) or aspartate aminotransferase (AST) > 2000 IU/mL within the first 7 postoperative days. According to the criteria of Verhoeven C et al. (34), the definition of ischemic-type biliary lesions (ITBL) in this study includes (i) intrahepatic or hilar bile duct(s) strictures and dilatation after OLT, which (ii) were confirmed by cholangiography and in the absence of hepatic artery thrombosis as demonstrated by Doppler ultrasonography, and (iii) which required endoscopic or percutaneous management in the biliary tract or liver retransplantation in recipients. Postoperative rejection was diagnosed according to the histological assessment.

### Sample Collection and Processing

All graft livers were procured following a standard procurement protocol (32, 34). After procurement and initial flush, the graft liver was stored in cold University of Wisconsin (UW) solution (Viaspan, Duramed Pharm Inc, Pomona, NY) or histidine tryptophan ketoglutarate (HTK) solution (Custodial HTK, Essential Pharmaceuticals, LLC, Pennsylvania, USA), and then transported to our center. Upon the arrival of the liver, a conventional back table procedure was performed by the surgeon. Briefly, an additional *ex situ* perfusion of the portal venous system was performed *via* gravity with 1000 ml of UW or HTK. A secondary flush was done under normal hydrostatic pressure with 500 ml of 4% human albumin solution (Albuman, Sanquin, The Netherlands), immediately before implantation. Liver biopsies were obtained at the end of the back table procedure (T0) and ~1 hour after portal reperfusion (T1).

### Rat Liver Transplantation Model

An established rat orthotopic liver transplantation method, which was approved by the institutional review board and the animal procedures, was conducted at the Laboratory of Experimental Surgery and Transplantation, Institute of Research Experimental and Clinic at the University Catholic of Louvain, Brussels, Belgium. For standardization and reproducibility purposes only male rats were used. Additionally, we need to infuse fluids intravenously through the penile vein during liver transplantation. Therefore male Lewis rats, purchased from Janvier Labs (Le Genest-Saint-Isle, France), weighing between 200 and 250 g at the time of the transplantation, were used as donors and recipients. Organ procurement and liver implantation were performed according to the full-vascularized technique, which included graft re-arterialization, previously described by Aiyakhagorn et al. (35). Rat livers (n=4) were procured and stored cold in Belzer UW^®^ preservation solution (Bridge to LifeTM – IL - USA) for 22 h before implantation. Before implantation, liver grafts were rinsed with 10 ml of 0.9% saline through the portal vein. Anastomosis of the suprahepatic vena cava, the portal vein, and the intrahepatic vena cava was performed with continuous suture using 7/0 polypropylene (Prolene^®^ - Ethicon - OH – USA) for the first and 8/0 for the last two. Graft arterialization was performed using a plastic stent telescoped into the donor celiac trunk and the recipient’s common hepatic artery. Biliary reconstruction was performed using a segment of 8 mm of a venous catheter 22g (Becton, Dickinson, and Company – NJ – USA) telescoped into the donor and recipient common bile duct and secured with 7/0 silk ligatures. Euthanasia was performed 24 hours after graft implantation for samples collection. Sham operations were conducted on rats (n=4) by performing a midline laparotomy under the same conditions as the liver recipients. Samples were collected 24 hours after the procedure.

### Histological Analyses

The liver specimens were routinely fixed with 4% paraformaldehyde for 24 hours, embedded in paraffin, and cut into 4µm sections. Sections were stained with hematoxylin and eosin (H&E) according to standard procedures. Immunostaining was performed as previously described (27). In short, sections were dewaxed and rehydrated *via* gradient ethanol washes. Antigen retrieval was then performed by heating the sections at 100°C in 10mM citrate acid buffer (pH 6.0). Incubation with 1% bovine serum albumin (BSA) and 10% normal goat serum (both from Sigma-Aldrich) in phosphate-buffered saline (PBS) were performed to prevent a-specific staining. The sections were then incubated with pMLKL (Thermofisher) antibody diluted in 1% BSA/0.025% Triton X100/1% normal goat serum (antibody diluent), at 4°C overnight. Information on all the primary antibodies used is listed in **Table S2**. Irrelevant rabbit IgG (Thermofisher) was applied as a negative isotype control. For immunohistochemistry, sections were incubated with 0.3% hydrogen peroxide for 15 min and by a 1-hour incubation with secondary goat anti-rabbit Immunoglobulins/HRP (Dako, Glostrup, Denmark) at room temperature. The reaction products were visualized using a 3,3’-Diaminobenzidine (DAB) substrate kit (Dako). Subsequently, the slides were counterstained with Mayer’s s hematoxylin and mounted in Pertex mounting medium. Whole-slide immunohistochemistry images were acquired on NanoZoomer (Hamamatsu, Iwata City, Japan).

Automated multiplex immunofluorescent staining was further performed using the Ventana Benchmark Discovery (Ventana Medical Systems Inc.) to determine the markers co-expressed with pMLKL. In brief, following deparaffinization and heat-induced antigen retrieval with CC1 (#950-500, Ventana) for 64 minutes at 97°C, the tissue samples were incubated with primary antibodies at 37°C in a step-by-step manner. The antibody denature step was performed between every antibody incubation and visualization using CC2 (#950-123, Ventana) for 20 minutes at 100˚C. pMLKL was incubated for 32 min, detected with Universal HQ kit (#760-275, Ventana), and visualization with R6G (#760-244, Ventana) for 4 minutes. CD90 was incubated for 60 minutes, detected with a Universal HQ kit, and visualized with DCC (#760-240, Ventana). Fibulin-2 was incubated for 32 minutes, detected with Universal HQ kit (#760-275, Ventana), and visualized with Red610 (#760-245, Ventana). CD45 was incubated for 32 minutes, detected with omnimap anti-mouse HRP (#760-4310, Ventana), and visualized with Cy5 (#760-238, Ventana) for 4 minutes. CD34 was incubated for 32 minutes, detected with omnimap anti-mouse HRP, and visualized with FAM (#760-243, Ventana) for 4 minutes. Slides were incubated in PBS with DAPI for 15 minutes and covered with an anti-fading medium (DAKO, S3023). Slides were scanned using the ZEISS Axio Imager 2.0 and analyzed using Qupath.

Information on the antibodies used can be found in **Table S2**.

### Immunohistochemistry Scoring

Immunohistochemistry scoring of pMLKL staining was performed electronically using the software ImageJ (imagej.nih.gov/ij) and the whole procedure was shown in **Figure S1A**. Considering pMLKL positivity was predominantly detected in the portal triad, at least 8 high-power fields (200X magnification) of portal triad areas were blindly selected for each section from the whole-slide images (representative images are shown in **Figure S1B**). The related clinical data belonging to the images was unknown at that time. After color deconvolution, the pixel intensity of cytoplasmic pMLKL staining, visualized by DAB, was automatically determined using the plugin IHC Profiler (36–38) and ImageJ software, in which high-positive, moderate-positive, low-positive, and negative zones were calculated. The threshold assignment was encoded in the plugin and performed automatically (38). We calculated the H-Score [scale 0 to 300, exemplified images were shown in **Figure S1C**)] for each image based on the generated products of the percentage contribution of positive zones and the intensity of labeling (0=negative; 1 = weak positive; 2 = moderate positive; 3 = high positive) using the following equation (36, 37, 39):

H-score= (percentage contribution of high-positive zone×3) + (percentage contribution moderate-positive zone×2) + (percentage contribution of low-positive zone)

Median H-scores in the selected fields were designated as the pMLKL positivity for each section. The pMLKL index for each graft liver was calculated by dividing the H-score in the T1 sample by the H-score in the T0 sample.

### Statistical Analysis

Statistical analysis was performed using SPSS statistics 25 (SPSS Inc, Chicago, IL, USA) and Prism software (GraphPad Software Inc., San Diego, USA). Data were presented as the median and interquartile range (IQR). Group comparisons were performed using the Mann-Whitney U test or Wilcoxon matched-pairs signed-rank test for continuous values and the chi-square test for categorical data. Spearman’s rank correlation test was conducted to estimate the linear relationship between variables. To identify risk factors for EAD, a logistic regression model was applied for multivariate analysis. A *p*-value of <0.05 was considered significant.

## Results

### MLKL Phosphorylation in Rat Hepatic IRI

**Figure 1:**
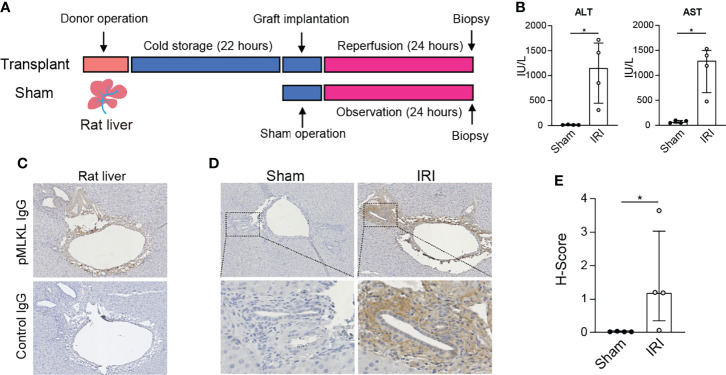
Expression of pMLKL in graft livers from the rat LT model. **(A)** Schematic representation of the timeline. Rat livers were procured and stored in cold preservation solution for 22 hours and then transplanted. Liver biopsies (n=4) were taken after 24 hours of reperfusion. **(B)** Serum ALT and AST in recipient rats after 24 hours of reperfusion were determined. The levels of both ALT and AST increased significantly in the IRI group compared with sham-operated rats. **(C)** Immunohistochemistry was performed on collected rat liver after IRI. Serial sections were stained with either pMLKL or a negative isotype control antibody. Shown is a representative microscopic image (magnification, 100X) confirming the specificity of the pMLKL antibody and the secondary antibody. **(D)** Representative microscopic images of pMLKL staining on rat livers undergoing either IRI or sham operation were shown (magnification, 100X). Enlarged images from the boxed area are shown in the bottom panel (magnification, 400X). Clear pMLKL was detected in the portal triads of rat liver undergoing IRI, but not in the sham-operated rats. **(E)** The H-Score of pMLKL staining was compared in rat donor liver undergoing hepatic IRI or sham operation (Mann-Whitney test), in which a significant increase in the IRI group was observed. *p < 0.05.

To determine whether the activation of pMLKL is involved in rat hepatic IRI, rat liver biopsies were collected after 22 hours of cold storage and 24 hours of reperfusion or sham operation (**Figure 1A**), followed by immunohistochemistry staining of pMLKL using a validated antibody (27, 40, 41). Compared to sham-operated rats, a significant increase of serum ALT (1153.0 (441.5-1652.0) vs 10.0 (10.0-17.5), *p*=0.029) and AST (1294.0 (654.5-1497.0) vs 66.5 (47.0-94.0), *p*=0.029) levels was observed in rats after LT (**Figure 1B**). The pMLKL expression in the liver after IRI is shown in **Figure 1C**. No positive staining was observed in rat liver biopsies incubated with negative isotype control antibody, confirming the specificity of the pMLKL and secondary antibodies. As shown in **Figure 1D**, The pMLKL positivity was barely observed in rat livers collected after the sham operation, suggesting that the increased expression of pMLKL was possibly associated with IRI. Clear pMLKL expression is detected in the portal triads but not in the liver parenchyma. As shown in **Figure 1E**, automated quantification of the histological score (H-Score) of pMLKL staining showed a significant increase in the IRI group versus sham group (1.524(1.80) *vs* 0.014 (0.01), *p*=0.029).

### Post-Reperfusion pMLKL Score Correlates With The Development of EAD After LT

**Figure 2:**
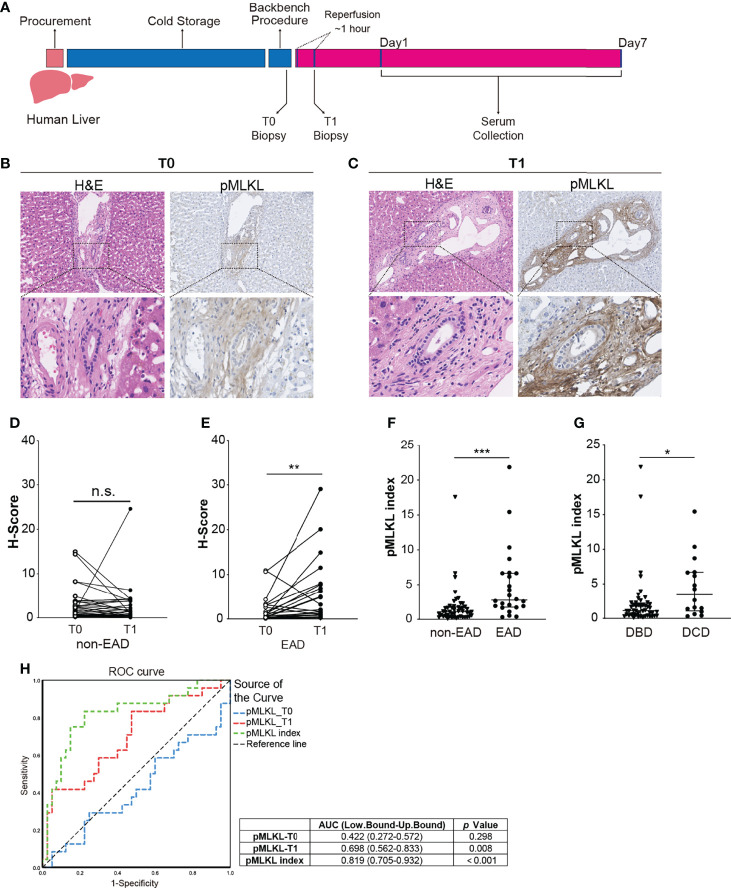
Periportal pMLKL expression in human LT biopsies. **(A)** Human graft livers were procured and stored in cold preservation solution following a conventional protocol. Liver biopsies were collected at the end of the backbench procedure (T0) and 1 hour after reperfusion (T1). Postoperative serums were collected in the first 7 post-LT days. **(B, C)** Representative microscopic images of HE and pMLKL staining on T0 and T1 graft livers that developed post-transplant EAD were shown (magnification, 100X). Enlarged images from the boxed area are shown in the bottom panel (magnification, 400X). The pMLKL positive cells were mainly localized at the periportal region, distributed within the stroma of the portal triad adjacent to the wall of the portal vein, hepatic artery, and biliary epithelium. **(D, E)** Paired comparison of pMLKL scores at T0 and T1 were performed separately in non-EAD and EAD groups (Wilcoxon matched-pairs signed-rank test). pMLKL-T1 was significantly higher than pMLKL-T0 in recipients that developed EAD compared to the non-EAD group. **(F)** pMLKL index were compared in non-EAD and EAD groups (Mann-Whitney test). The pMLKL index was significantly higher in the EAD group versus the non-EAD group. **(G)** pMLKL index were compared in DBD and DCD livers. (Mann-Whitney test). A significantly higher pMLKL index was observed in DCD liver grafts compared to the DBD livers **(H)** ROC curve showing the discriminative ability of pMLKL_T0, pMLKL_T1, and pMLKL index in the development of EAD. Data are presented as the median and interquartile range (IQR). n.s., not significant; *p < 0.05; **p < 0.01; ***p < 0.001.

To investigate the expression of pMLKL in human liver grafts, a retrospective study was performed (**Figure 2A**). Out of 64 included LT patients, 24 recipients developed EAD (38%). (**Table S1**). Representative H&E and pMLKL stained images of T0 and T1 biopsies are shown in **Figures 2B, C**. The pMLKL^+^ cells mainly had a periportal localization, distributed within the stroma of the portal triad adjacent to the wall of the portal vein, hepatic artery, and biliary epithelium. The paired analysis further showed that pMLKL-T1 was significantly higher than pMLKL-T0 in recipients that developed EAD (1.88 (0.85- 7.55) vs. 0.70 (0.21-2.97), *p*=0.008) rather than the non-EAD group (0.75 (0.35- 2.74) vs. 1.14 (0.37-2.78), *p*=0.289) (**Figures 2D, E**). The pMLKL index was significantly higher in the EAD group versus the non-EAD group (2.77 (1.75-6.65) vs. 0.95 (0.39-1.62), *p*<0.0001) (**Figure 2F**). Interestingly, also a significantly higher pMLKL index was observed in DCD liver grafts compared to DBD livers (3.44 (1.06-6.71) vs. 1.10 (0.42-2.21), p=0.019) (**Figure 2G**). Moreover, based on the receiver operating characteristic (ROC) curve, the pMLKL index outperformed the pMLKL-T1 in predicting the development of EAD (**Figure 2H**). We further found an optimal cut-off value of 1.64 of the pMLKL index displaying the most optimized sum of sensitivity (83.3%) and specificity (77.7%).

**Figure 3:**
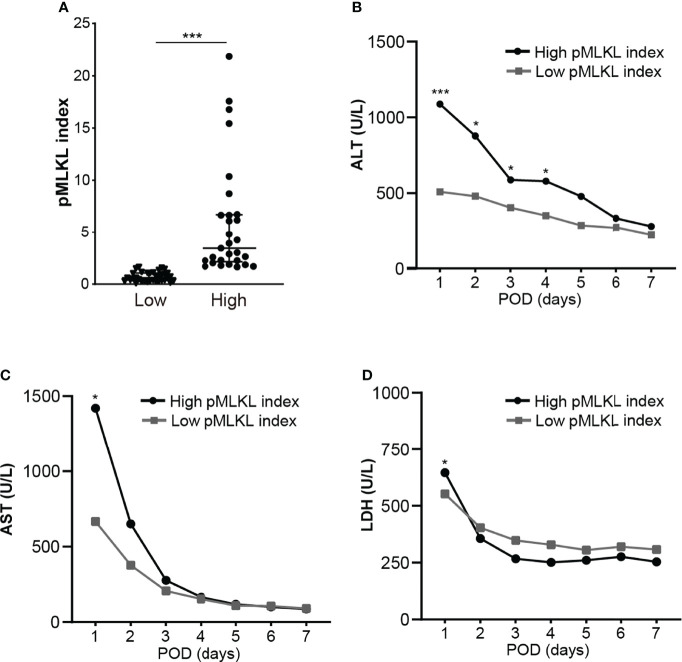
The histological score of pMLKL correlates with early liver injury after reperfusion. **(A)** The dichotomy of graft pMLKL index is grouped as low and high. Recipients were classified into low (≤1.64) versus high (>1.64) pMLKL index. **(B, C, D)** Levels of serum ALT, AST, and LDH at POD1-7 were compared (median, two-way ANOVA test followed by Sidak’s multiple comparisons test). The high pMLKL index group had significantly higher serum ALT/AST/LDH levels on POD1. Data are presented as the median and interquartile range (IQR). *p < 0.05; ***p < 0.001.

Based on the cut-off value of the pMLKL index, 64 grafts were allocated to a low pMLKL index (<1.64; n=35) and a high pMLKL index (≥1.64; n=29) group. Demographics of donors and recipients with low pMLKL index grafts versus high pMLKL index grafts are shown in **Table 1**. The high-pMLKL index group showed a higher trend in the last serum AST of the donor before procurement. Interestingly, a higher portion of ITBL occurrence was observed in the high-pMLKL index group though the difference was not significant. The distribution of the pMLKL index in high and low groups is shown in **Figure 3A**. As shown in **Figure 3B**, the high pMLKL index group had a significantly higher serum ALT level at POD1-4 (*p*<0.05). Also, the AST level at POD1 (**Figure 3C**, *p*<0.05), and LDH level at POD1 (**Figure 3D**, *p*<0.05) were elevated in this group. Correlation analysis based on the entire cohort revealed a relatively weak correlation between serum ALT at POD1 and pMLKL index (rs=0.400, p<0.01) and between serum AST at POD1 and pMLKL index (rs=0.312, p<0.05) (data not shown).

**Table 1:** Donors and recipients characteristics of liver grafts with low pMLKL index versus high pMLKL index.

	Low pMLKL index (n=35)	High pMLKL index (n=29)	p value
**Donor characteristics**			
Donor type (DCD)	7 (20%)	9 (31%)	0.234
Age (yr)	60 (26.0)	53 (13.0)	0.235
Sex (male)	18 (51%)	16 (55%)	0.274
BMI (kg/m2)	23.9 (4.3)	23.2 (4.0)	0.952
Last AST (U/l)	41.0 (30.0)	51.0 (35.5)	0.074
Last ALT (U/l)	25.0 (38.0)	34.0 (34.5)	0.395
Donor risk index	2.0 (0.8)	2.0 (3.88)	0.700
warm ischemia time (min)	27.0 (10.0)	26.0 (11.0)	0.787
cold ischemia time (min)	403.0 (118.0)	369 (109.0)	0.187
**Recipient characteristics**			
Age (yr)	46 (21.0)	53 (14.0)	0.040*
Sex (male)	16 (46%)	12 (41%)	0.146
Lab-Meld score	24.0 (7.0)	26.0 (15.0)	0.855
Transplantation indication			
-Autoimmune hepatitis	16 (46%)	12 (41%)	0.463
-Alcohol	2 (6%)	4 (14%)	0.250
-Hepatitis B/C	6 (17%)	7 (24%)	0.351
-Hepatocellular carcinoma	3 (9%)	9 (31%)	0.024*
-Other	9 (26%)	5 (17%)	0.306
Anastomosis ductus (Roux-Y)	5 (14%)	8 (28%)	0.140
**Post-transplantation complication**			
EAD	4 (11%)	20 (69%)	<0.001*
Hepatic artery thrombosis	4 (11%)	1 (3%)	0.242
Rejection	5 (14%)	6 (21%)	0.364
ITBL	7 (20%)	12 (41%)	0.056
AKI	6 (17%)	4 (14%)	0.454

*Significance.

### pMLKL Index Is a Predictor Factor for EAD

To determine whether the pMLKL-T1 score or pMLKL index are independent predictors for EAD, we conducted a multivariate analysis (**Table 2**). Univariate analysis indicates the EAD risk factors in this cohort, including higher pMLKL-T1 score (1.88 (0.85- 7.55) vs. 0.75 (0.35- 2.74), *p* = 0.059), higher pMLKL index, DCD grafts (38% vs. 18%, *p*=0.079) and male donors (67% vs. 45%, *p*=0.096) (**Table S1**). On multivariate analysis, only the high pMLKL index was identified as an independent predictive factor for EAD (odds ratio = 1.348; 95% CI, 1.066-1.703; *p* = 0.013). In this model, the discriminative power of the pMLKL index is indicated as a C-statistics calculation that reached up to 0.932 and was not below 0.705.

**Table 2:** Univariate and multivariate analysis for risk factors for post-transplant EAD.

Factors	OR (95% CI)	Univariate		Multivariate	
		*p* value	OR (95% CI)	*p* value	C-statistics (low. limit-up. limit)
DCD graft	0.354 (0.111-1.129)	0.079	-	0.473	-
Male donor	0.409 (0.143-1.172)	0.096	-	0.064	-
pMLKL-T1	1.133 (0.995-1.290)	0.059	-	0.472	-
pMLKL index	1.347 (1.067-1.700)	0.012	1.348 (1.066-1.703)	0.013	0.819 (0.705-0.932)

OR, Odds ratio.

### Activation of pMLKL in Multiple Non-Parenchymal Cells in the Portal Triad

**Figure 4:**
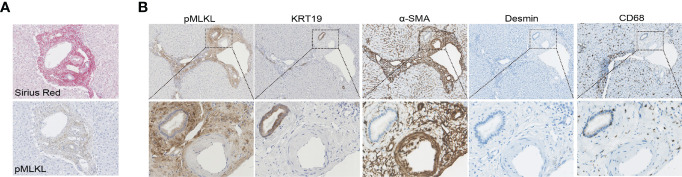
Characterization of periportal cells expressed pMLKL. **(A, B)** Serial liver sections from T1 biopsies obtained from one donor liver were stained with Sirius Red, pMLKL, KRT19, α-SMA, Desmin, and CD68 (magnification 100X). Enlarged images from the boxed area are shown in the bottom panel (magnification 400X). ECM deposition was observed mostly in the periportal area and appeared to overlap with pMLKL positivity. Areas positive for pMLKL overlapped a lot with α-SMA^+^, but not Desmin^+^. Part of KRT19^+^ cells also displayed positive staining for pMLKL, while few CD68^+^ were observed to co-express pMLKL.

Considering periportal non-parenchymal cells seem to be the major source of pMLKL^+^ cells, we aim to characterize the cellular populations in portal triads with pMLKL positivity. ECM deposition, demonstrated by Picro Sirius Red staining, was observed mostly in the periportal area, overlapping with pMLKL positivity (**Figure 4A**). We reasoned that hepatic IRI might activate pMLKL predominantly in the myofibroblast after a short period of reperfusion. To this end, immunohistochemistry on serial sections for multiple markers was performed to identify the populations of periportal cells including cholangiocyte (KRT19), activated myofibroblast [α-smooth muscle actin (α-SMA)], hepatic stellate cells (HSCs) (Desmin), and macrophage (CD68) (**Figure 4B**). We found that area positive for pMLKL partially overlapped with α-SMA^+^ cells, suggesting that myofibroblasts might be one of the cell types co-expressing pMLKL. The absence of Desmin^+^ cells overlapping with pMLKL expression reveals that the myofibroblasts co-expressing pMLKL might be not derived from HSCs. Interestingly, part of biliary epithelial cells labeled with KRT19 also displays positive staining for pMLKL. CD68 staining further indicated that macrophages were not the predominant cells comprising the pMLKL^+^ population.

**Figure 5:**
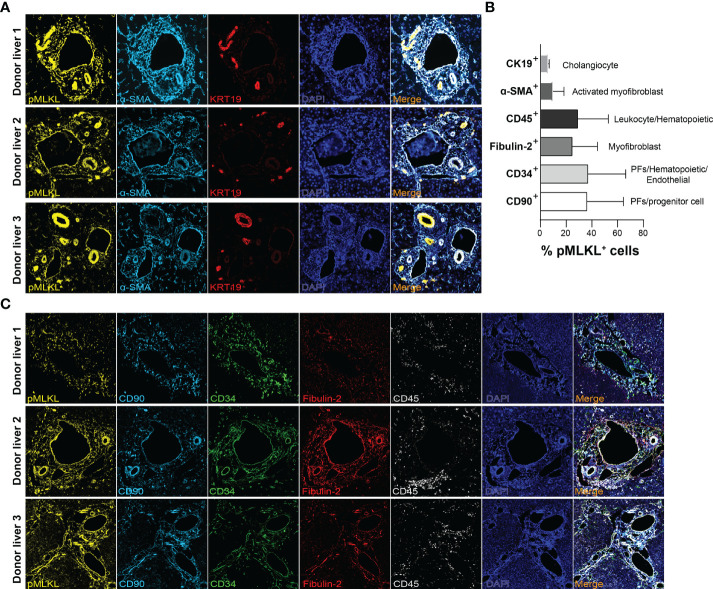
Characterization of periportal cells expressed pMLKL. **(A, B)** Multiplex immunofluorescent staining was performed on T1 graft livers (n=3) for pMLKL, α-SMA, KRT19, CD90, CD34, Fibulin-2, and CD45 (magnification, 400X). **(C)** The composition of pMLKL+ cells (100%) was calculated as the percentage of cells co-expressed with multiple markers (mean ± SD). The periportal non-parenchymal cells and cholangiocytes predominantly constitute the population co-expressing pMLKL.

**Figure 6:**
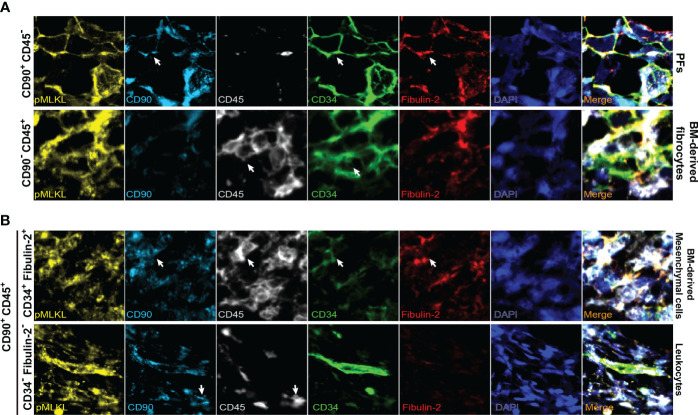
Representative multiplex immunofluorescent images of distinct subpopulations of pMLKL expressed cells in portal triads (magnification, 2000X). Arrows indicate the cells with positive staining. The pMLKL^+^ cells featured by CD90^+^ CD45^-^ (PFs), CD90^-^ CD45^+^ (BM-derived fibrocytes) **(A)**, CD90^+^ CD45^+^ CD34^+^ fibulin-2^+^ (BM-derived mesenchymal cells) and CD90^+^ CD45^+^ CD34^-^ fibulin-2^-^ (leukocytes) **(B)** were shown.

To support our findings, we performed multiplex immunofluorescent staining on the biopsies collected from 3 human donor livers after perfusion to further determine the co-localization of pMLKL^+^ cells with periportal cells. Likewise, we found KRT19^+^ cholangiocytes constitute a small population of pMLKL^+^ cells (5.92 ± 0.88%) (**Figures 5A, B**). It has been reported that CD90, CD34, and fibulin-2 represent the critical, but not exclusive, markers for liver myofibroblast residing near portal vein during chronic injury (30, 31). The periportal cells expressing CD90 (36.15 ± 28.4%), CD34 (36.9 ± 29.4%), and fibulin-2 (29 ± 23.79%) predominantly overlayed with pMLKL^+^ cells in the portal triad (**Figure 5C**). Given that CD90 and CD34 could also be expressed on the surface of multiple cell types separately, such as lymphocytes and bone marrow-derived fibrocytes, we applied CD45, a leukocyte/hematopoietic marker, staining to further characterize the subpopulation of periportal pMLKL^+^ cells. A large number of pMLKL^+^ cells co-expressing CD90, CD34, and Fibulin-2 were accumulated in the portal triad and designated as PFs (**Figure 6A**) and exhibited a fibroblast-like spindle shape. On the other hand, a small number of pMLKL^+^ CD90^-^ cells co-expressed CD45 and CD34 were observed, indicating that these cells might be bone marrow-derived fibrocytes (30). Notably, we also found a few pMLKL^+^ CD90^+^ CD45^+^ cells which could be separated into two distinct subpopulations including CD34^+^ fibulin-2^+^ cells (bone barrow-derived mesenchymal cells) and CD34^-^ fibulin-2^-^ cells (leukocytes) (42) (**Figure 6B**). Collectively, we concluded that necroptosis executor pMLKL could be activated by hepatic IRI predominantly in periportal non-parenchymal cells including PFs, fibrocytes, and a minority of cholangiocytes.

## Discussion

In this study, we demonstrated that the pMLKL expression increased significantly in the portal triads after reperfusion in both human and rat LT. The pMLKL index based on histological assessment correlates with early liver injury after LT and is predictive of post-transplant EAD. We further confirmed that activation of pMLKL occurs in multiple periportal non-parenchymal cells and cholangiocytes. To the best of our knowledge, our study is the first to investigate the clinical relevance of the necroptosis mediator in human liver transplantation.

Cell death is an eventual event in IRI during liver transplantation and is strongly linked to the short- and long-term outcomes of recipients. In the last decades, mitigating cell death represents one of the most investigated strategies to improve organ functionality and minimize post-transplant complications (43). In previous studies, apoptosis has been one of the key targets, but the translation into clinical practice has been merely lacking. Except for apoptosis, the emerging role of programmed cell death has been validated in hepatic IRI, expanding our knowledge of the different cell death programs during LT. We previously reported that most pMLKL positive cells were found in the portal triads in LT biopsies, but were absent in ischemia-free liver biopsies collected from living donors grafts, suggesting the involvement of pMLKL-mediated cell death in hepatic IRI (27). Myofibroblasts are the main effectors of liver fibrosis (31) and we concluded in this study that myofibroblasts represent a major cell type with pMLKL activation. In fact, the periportal expression pattern of pMLKL and its activation in myofibroblast was barely found in liver biopsies obtained from patients with end-stage liver diseases, which were generally featured by severe liver fibrosis and extensively activated fibroblast (27). This discrepancy may imply that the activation of pMLKL during liver transplantation represents an event with a unique mechanism that is different from other chronic etiologies. Besides, in this study, immunostaining on both pre-and post-reperfusion biopsies further revealed an increase in pMLKL expression at T1 compared to T0. This suggests that during graft reperfusion extensive pMLKL-mediated cell death is possibly induced and therefore this might be the right time to mitigate cell death.

Considering that cells expressing pMLKL were mostly detected in the portal triad, we only selected the periportal areas for the analysis. These areas account for a much smaller portion of the liver tissue compared to the parenchymal regions. We assume that this method provides a more precise score of periportal pMLKL expression. Interestingly, the pMLKL score appears to be associated not only with EAD but also with ITBL though the difference was not significant. It is well known that the liver cells, including hepatocytes and cholangiocytes, that are damaged during hepatic IRI, often direct the onset of EAD or ITBL. Our previous studies demonstrated that the release of cholangiocyte-derived microRNAs predicted the development of ITBL (34), while the hepatocyte-derived microRNAs predicted the occurrence of EAD (32). In this study, we found that during LT, pMLKL could be activated in cholangiocytes. This might be a potential cause of biliary injury during LT and possibly contributes to the high incidence of ITBL in our EAD cohort. In addition, the pMLKL index correlated with the serum levels of ALT and AST, often released from necrotic hepatocytes, on POD1, but not POD7 (data not shown). Given that low levels of pMLKL expression were observed in the liver parenchyma, we speculate that the periportal necroptosis in nonparenchymal cells could have a detrimental effect on hepatocytes indirectly, though the exact mechanism is still unclear.

We further characterized the pMLKL-expressing cells and found that next to cholangiocytes, also non-parenchymal cells such as periportal mesenchymal cells, including PFs and fibrocytes, were mainly stained with pMLKL. In the adult liver, myofibroblasts represent the main effectors of fibrous stress during liver injury by regulating the wound healing response, including liver repair and regeneration (31). Upon this stress factor, liver mesenchymal cells, including HSCs, PFs, and fibrocytes can differentiate into myofibroblasts, depending on the mechanism of the insults. We speculate that myofibroblasts that expressed pMLKL, share similar signatures with PFs and fibrocytes, but not with HSCs. The pMLKL activation in myofibroblast and its relationship with hepatic IRI has been poorly investigated. In previous studies, activated PFs have been reported to contribute to fibrous scar production in chronic cholestatic fibrosis (30). Besides, a recent retrospective study revealed that histological scores of collagen deposition in donor livers at the time of procurement, correlated with an increased incidence of post-transplant EAD, suggesting an unexpected role of collagen-producing cells in hepatic IRI (44). Konishi et al. (45) also reported that fibrotic livers show accelerated recovery and repair after hepatic IRI compared to the normal liver in experimental models. Although the precise mechanisms of how pMLKL was activated in myofibroblasts during LT remain to be determined, we envision that the massive necroptosis of myofibroblasts residing in the fibrotic liver before LT, could be induced by short-time reperfusion, which might lead to an imbalance of hepatic damage and repair, finally resulting in “irreversible” IRI in graft liver. This could be led by impairment of liver regenerative capacity due to myofibroblast depletion or secondary necroinflammation mediated by necroptotic myofibroblasts. The interaction among hepatic IRI, myofibroblasts, and necroptosis is worth further investigation.

It is important to note that the necroptosis mediators, such as MLKL and RIPK3, could exert multifactorial functions which could be linked to other types of programmed cell death or even be independent of cell death (46, 47). For instance, we have previously reported that nuclear pMLKL was expressed in TNF-a-induced apoptosis in cholangiocyte organoids (27). Given that cytoplasmic and membrane translocation of pMLKL is widely regarded as the major functional form of MLKL for necroptosis execution, the nucleus-located pMLKL in apoptotic cholangiocyte organoids might suggest an event possibly serving a unique role independent of necroptosis. Cao et al. (18) demonstrated that nuclear MLKL represents a contributor to endoplasmic reticulum stress-related apoptosis, which might be a potential elucidation. In the present study, we observed distinct expression patterns of pMLKL in different liver cell types, in which both cytoplasmic/membrane and a minority of nuclear pMLKL were confirmed. This suggests that the increased expression of pMLKL during hepatic IRI might be invoked by multiple mechanisms and leads to distinct outcomes. We think it is important to reach a consensus on the definition of necroptosis, which is still lacking. Given that the interactions of MLKL with other cell death-dependent or -independent pathways are emerging, the cytoplasmic/membrane staining of pMLKL is not enough to define necroptotic cell death. In addition to pMLKL detection, ultrastructural examination using transmission electron microscopy analysis might be helpful to further determine the morphological change of dying cells and characterize the exact cell death type.

Our knowledge of the regulated necrosis network, such as necroptosis, ferroptosis, and pyroptosis is expanded in the last years. Notably, the expression of the mediators of multiple cell death programs differs among parenchymal and non-parenchymal liver cells at the transcriptional and protein levels, which potentially determines the cell death modalities in different types of cells upon certain stress factors (15, 48, 49). In our study, pMLKL was predominantly activated in cholangiocytes and nonparenchymal (stromal) cells. It has also been reported that pyroptosis induced in macrophages aggravates hepatic IRI by promoting an inflammatory response in experimental models (50, 51). Yamada N et al. demonstrated that an increase of ferroptosis markers in serum is a risk factor for human LT, and inhibition of ferroptosis prevents the infiltration of neutrophils and macrophages in the murine liver (52). Taken together, the multiple regulated necrosis modalities may contribute to hepatic IRI in a cell type-specific manner. Whether these regulated necrosis programs promote hepatic IRI independently or synergistically remains unclear. Future investigations should be performed based on human materials and animal models and using single-cell profiling methods including both transcriptomes and proteomes.

It is important to note that there are obvious limitations in our study, mostly because of the relatively small cohort and limited availability of multiple types of specimens. For instance, it remains a challenge to characterize the cells co-expressing pMLKL in clinical biopsies only in a histological manner. In this retrospective study, we were not able to obtain fresh biopsies to perform flow cytometry, which is supposed to be a better method for cellular populations identification. Based on an appropriate biopsy collection, future prospective studies should be performed including flow cytometry assessment. Besides, it would be interesting to further identify the distinct cellular populations using lineage tracing in experimental IRI models. Moreover, although we confirmed a significant increase in pMLKL scores after short-time reperfusion, we also observed strong pMLKL positivity in a few T0 biopsies, indicating that there might also be baseline injury or preservation damage in these donor’s livers. Based on our study, we cannot conclude whether pMLKL has already been activated before procurement or could be induced or potentiated by cold preservation. This is obviously due to the lack of pre-preservation baseline biopsies. To address this, biopsies obtained at different time points during liver transplantation should be investigated in the future.

In conclusion, our study reveals that pMLKL expression increased significantly after reperfusion in both rodent and human LT. The histological score of pMLKL is predictive of post-transplant EAD and is associated with early liver injury after LT. Periportal non-parenchymal cells appear most susceptible to pMLKL-mediated cell death during hepatic IRI.

## Data Availability Statement

The raw data supporting the conclusions of this article will be made available by the authors, without undue reservation.

## Ethics Statement

The studies involving human participants were reviewed and approved by Erasmus MC medical ethics council. The patients/participants provided their written informed consent to participate in this study. The animal study was reviewed and approved by Université catholique de Louvain.

## Author Contributions

SS, EB-R, IS, MV, and LL contributed to the conception and design of the study. SS, IS, and HR organized the database. SS, IS and KL performed the statistical analysis. SS wrote the first draft of the manuscript. EB-R, IS, and TB wrote sections of the manuscript. All authors contributed to manuscript revision, read, and approved the submitted version.

## Funding

This study was partly funded by the Medical Delta program grant (Regenerative Medicine 4D) and TKI-LSH (Topconsortium Kennis en Innovatie-Life Sciences & Health) grant (RELOAD, EMC-LSH19002). SS is financially supported by the China Scholarship Council (No. 201706230252).

## Conflict of Interest

The authors declare that the research was conducted in the absence of any commercial or financial relationships that could be construed as a potential conflict of interest.

## Publisher’s Note

All claims expressed in this article are solely those of the authors and do not necessarily represent those of their affiliated organizations, or those of the publisher, the editors and the reviewers. Any product that may be evaluated in this article, or claim that may be made by its manufacturer, is not guaranteed or endorsed by the publisher.

## Supplementary Material

Supplementary Table 1 Donor and recipient characteristics.

Supplementary Table 2 List of antibodies used in this study.
