# Supplementary material for: Liver Ischemia and Reperfusion Induce Periportal Expression of Necroptosis Executor pMLKL Which Is Associated With Early Allograft Dysfunction After Transplantation
Source: Front Immunol. 2022 May 17;13:890353. doi: 10.3389/fimmu.2022.890353 (PMC9152120; doi:10.3389/fimmu.2022.890353)
Supplement: Supplementary Table 1 — Donor and recipient characteristics. [file DataSheet_1.docx]

Table S1. Donor and recipient characteristics.

|  | Non-EAD (n=40) | EAD (n=24) | p value |
| --- | --- | --- | --- |
| Donor characteristics | | | |
| Donor type (DCD) | 7 (18%) | 9 (38%) | 0.079 |
| Age (yr) | 59 (17.8) | 50 (13.5) | 0.255 |
| Sex (male) | 18 (45%) | 16 (67%) | 0.096 |
| BMI (kg/m2) | 24.2 (4.9) | 23.1 (2.7) | 0.364 |
| Cause of death |  |  |  |
| -Trauma | 7 (18%) | 3 (13%) | 0.594 |
| -Cerebrovascular accident | 28 (70%) | 16 (67%) | 0.781 |
| -Anoxia | 1 (3%) | 2 (8%) | 0.2851 |
| -Other | 4 (10%) | 3 (13%) | 0.756 |
| Last AST (U/l) | 45.0 (30.0) | 47.5 (32.5) | 0.298 |
| Last ALT (U/l) | 28.0 (40.5) | 33.0 (30.0) | 0.328 |
| warm ischemia time (min) | 27.0 (10.0) | 26.0 (7.7) | 0.264 |
| cold ischemia time (min) | 371.5 (120.0) | 384.5 (118.0) | 0.339 |
| Recipient characteristics | | | |
| Age (yr) | 50 (16) | 52 (15) | 0.289 |
| Sex (male) | 24 (60%) | 12 (50%) | 0.435 |
| Lab-Meld score | 24.0 (7.5) | 29.0 (6.1) | 0.840 |
| Transplantation indication | | | |
| -Autoimmune hepatitis | 18 (45%) | 3 (13%) | 0.501 |
| -Alcohol | 3(8%) | 10 (42%) | 0.402 |
| -Hepatitis B/C | 8(20%) | 5 (21%) | 0.589 |
| -Hepatocellular carcinoma | 5 (8%) | 7 (29%) | 0.094 |
| -Other | 10 (25%) | 4 (17%) | 0.325 |
| Anastomosis ductus (Roux-Y) | 6 (15%) | 8 (23%) | 0.051 |
| Laboratory results 24 hours after transplantation | | | |
| AST (U/l) | 798.5 (477) | 4882.0 (4602.0) | 0.0001 |
| ALT (U/l) | 652.0 (485.0) | 3259.0 (3107.0) | 0.0001 |
| LDH (U/l) | 727.5 (487.0) | 4364.0 (4256.0) | 0.0001 |
| Creatinine (umol/l) | 93.0 (87.0) | 10 (4256.0) | 0.703 |
| INR | 1.7 (1.0) | 2.1 (1.0) | 0.104 |
| Billirubin (umol/l) | 33.0 (16.0) | 25.5 (25.0) | 0.305 |
| Post-transplantation complication | | | |
| Hepatic artery thrombosis | 2 (5%) | 3 (13%) | 0.279 |
| Rejection | 8 (20%) | 3 (13%) | 0.441 |
| ITBL | 7 (18%) | 12 (50%) | 0.006 |
| AKI | 8 (20%) | 2 (8%) | 0.185 |

Table S2. List of antibodies used in this study.

| Antibody | Reactivity | Application | Species | | Source | Concentration |
| --- | --- | --- | --- | --- | --- | --- |
| pMLKL | Human/Rat | IHC/IF | Rabbit | Polyclonal | Invitrogen (PA5-105678) | 1:100 (IF)  1:200 (IHC) |
| KRT19 | Human | IHC | Mouse | Monoclonal | DAKO (M088801-2) | 1:50 |
| KRT19 | Human | IF | Rabbit | Monoclonal | Cell Marque (319R-24) | 1.4 µg/ml |
| CD34 | Human | IHC/IF | Mouse | Monoclonal | Abcam (ab8536) | 0.8 µg/ml |
| CD45 | Human | IHC/IF | Mouse | Monoclonal | Ventana (760-4279) | 0.8 µg/ml |
| CD90 | Human | IHC/IF | Mouse | Monoclonal | Novus (NBP2-37330) | 1:50 |
| CD68 | Human | IHC | Mouse | Monoclonal | Cell Marque (168M-96) | 1:100 |
| Fibulin-2 | Human | IHC/IF | Mouse | Polyclonal | Santa Cruz | 1:100 |
| α-SMA | Human | IHC/IF | Mouse | Monoclonal | Cell Marque (202M-94) | 1.4 µg/ml |
| Collagen I | Human | IF | Rabbit | Polyclonal | Novusbio (NB600-408) | 1:200 |
| Desmin | Human | IHC/IF | Mouse | Monoclonal | Cell Marque (243M-14) | 1.6 µg/ml |
