# Supplementary figures and images for: Liver Ischemia and Reperfusion Induce Periportal Expression of Necroptosis Executor pMLKL Which Is Associated With Early Allograft Dysfunction After Transplantation
Source: Front Immunol. 2022 May 17;13:890353. doi: 10.3389/fimmu.2022.890353 (PMC9152120; doi:10.3389/fimmu.2022.890353)

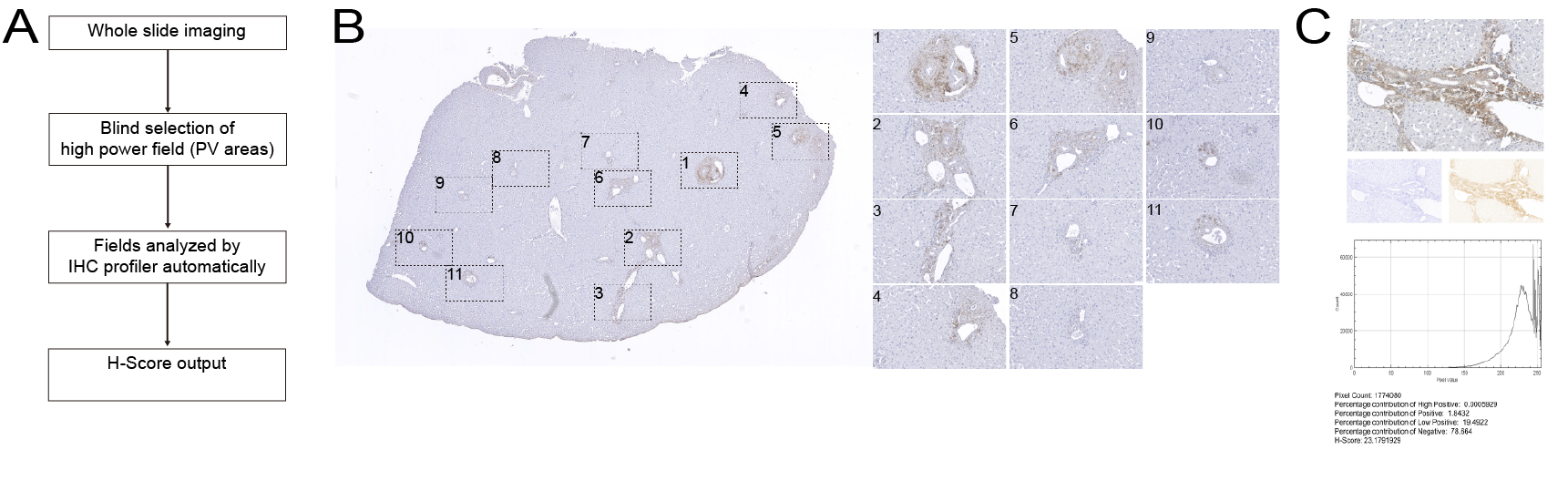

Supplement: Supplementary file 2 [file Image_1.tif]
